# An end-to-end deep learning framework for translating mass spectra to de-novo molecules

**DOI:** 10.1038/s42004-023-00932-3

**Published:** 2023-06-23

**Authors:** Eleni E. Litsa, Vijil Chenthamarakshan, Payel Das, Lydia E. Kavraki

**Affiliations:** 1 Department of Computer Science, Rice University, Houston, TX USA; 2 IBM Research, IBM Thomas J. Watson Research Center, Yorktown Heights, NY USA

**Keywords:** Cheminformatics, Metabolomics, Method development, Structure prediction

## Abstract

Elucidating the structure of a chemical compound is a fundamental task in chemistry with applications in multiple domains including drug discovery, precision medicine, and biomarker discovery. The common practice for elucidating the structure of a compound is to obtain a mass spectrum and subsequently retrieve its structure from spectral databases. However, these methods fail for novel molecules that are not present in the reference database. We propose Spec2Mol, a deep learning architecture for molecular structure recommendation given mass spectra alone. Spec2Mol is inspired by the Speech2Text deep learning architectures for translating audio signals into text. Our approach is based on an encoder-decoder architecture. The encoder learns the spectra embeddings, while the decoder, pre-trained on a massive dataset of chemical structures for translating between different molecular representations, reconstructs SMILES sequences of the recommended chemical structures. We have evaluated Spec2Mol by assessing the molecular similarity between the recommended structures and the original structure. Our analysis showed that Spec2Mol is able to identify the presence of key molecular substructures from its mass spectrum, and shows on par performance, when compared to existing fragmentation tree methods particularly when test structure information is not available during training or present in the reference database.

## Abstract (web summary)

Identification of chemical compounds, present in a sample, is a fundamental task of chemical analysis, but matching obtained spectra with chemical databases is limited to identifying known molecules. Here, the authors report a deep learning architecture for recommending molecular structures, including those of novel molecules, given sample mass spectra alone.

## Introduction

The identification of the chemical compounds that are present in a sample of chemical matter is a fundamental task in chemical analysis with applications in multiple domains. The field of metabolomics, for example, seeks to identify the chemical molecules that are present in a biological sample. In humans, the metabolome, that is the set of all chemical molecules that can be found in human tissues, is a great source for biomarker discovery as it reflects changes at a genetic, proteomic or environmental level^1^. Additionally, mapping the human metabolome will lead to a better understanding of human physiology and disease etiology and pathology which is essential for the identification of new therapeutic targets for developing new treatments. The increasing interest in mapping the metabolome extends to other organisms as well, such as plants which have been a great source of bioactive compounds for multiple products including drugs and supplements^2^. The identification of chemical compounds is also critical in product development such as in the production of pharmaceuticals and agrochemicals. Structure elucidation practices are being used for quality control and detection of impurities, as well as in safety studies for identifying potential metabolites that can be formed in the human body. Finally, structure elucidation practices are being employed in forensics analysis.

**Fig. 1:**
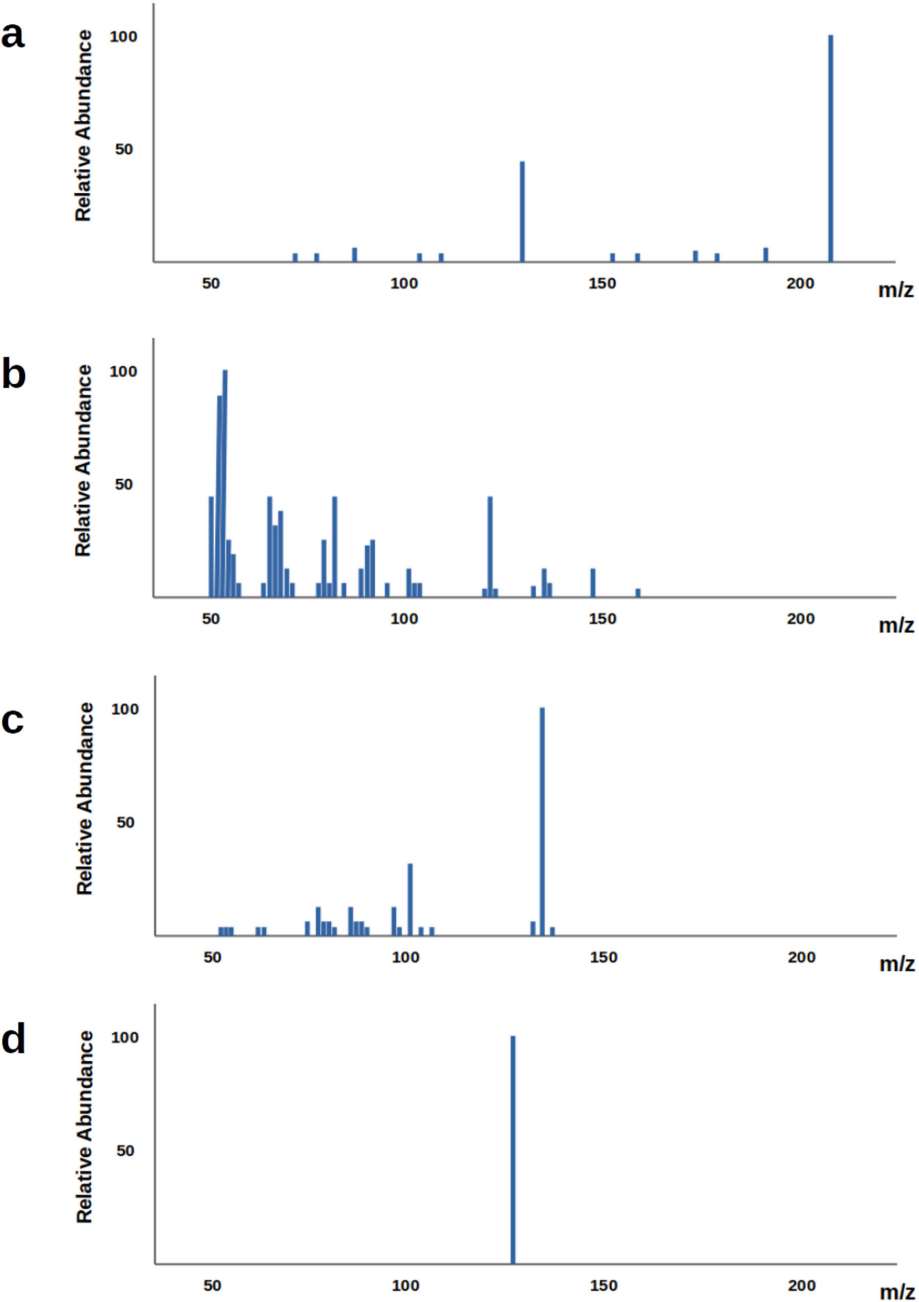
MS/MS spectra from different experimental conditions for the same molecule. MS/MS spectra obtained through different experimental conditions from the same molecule (approximate spectra based on data obtained from the Human Metabolome Database). **a** Precursor ion: [M+H]+, NCE: 35%, Instrument: HCD. **b** Precursor ion: [M+H]+, NCE: 130%, Instrument: HCD. **c** Precursor ion: [M+H-Br]+, NCE: 35%, Instrument: HCD. **d** Precursor ion: [M+H+2i]+, NCE: 35%, Instrument: IT-FT.

The identification of the structure of a chemical compound is perceived as one of the most time consuming and laborious task in chemical analysis. This is often performed through analytical techniques such as mass spectroscopy (MS) and nuclear magnetic resonance (NMR)^3–5^ with MS being used more often due to its higher sensitivity and specificity^3^. In MS, the molecules that are present in a biological sample are first separated using a chromatographic technique, such as liquid chromatography (LC) and gas chromatography (GC), with the latter being used more commonly^1,6^. After the separation, the molecule is fragmented into positive or negative charged ions using an ionization source such as electron ionization (EI), chemical ionization (CI) and electrospray ionization source (ESI)^1,6^. What the instrument records is the mass-to-charge (*m*/*z*) ratios of the generated fragment ions. The information that is collected from this process is presented in the mass spectrum which is a graph with the *m*/*z* of each recorded fragment in the horizontal axis and the relative abundance in the vertical axis. In order to obtain more detailed information on the query structure, a sequential fragmentation process is often used called tandem mass spectrometry^5^. Once the molecule has been fragmented into ions, a set of them, called precursor ions, is selected and further fragmented to generate MS2 (also called MS/MS) spectra. These second-level ions can be fragmented even further giving MS3 spectra and so on. The peaks and their intensity in the resulting spectrum depend not only on the structure of the chemical molecule that is being fragmented, but also on the experimental conditions, that is the instrument used, the collision energy, the selected precursor ion and the ionization mode, as it is illustrated in Fig. 1.

Once the mass spectrum is obtained, it is matched against the content of spectral databases of reference compounds in order to retrieve its structure. Various chemical databases provide spectra data of metabolites^7^ such as Human Metabolome Database, METLIN, MassBank, and mzCloud^7^. Certain databases are focused on the metabolites of specific organisms, such as the Human Metabolome Database, or on specific molecular classes, such as the LIPID MAPS Structure Database, while others have greater coverage such as METLIN. However, despite the intense ongoing efforts to map the metabolome of various organisms, existing databases cover only a small percentage of the actual metabolites that occur in organisms. Particularly for humans, it is estimated that less than 10% of metabolites have experimental reference mass spectra^8^, which means that the current practice cannot identify a large percentage of the molecules that are found in human tissues. It is estimated that in untargeted metabolomics studies less than 2% of the detected spectral features are identified^8^.

An approach that has been developed to address the problem of limited amount of experimental spectra data is in silico fragmentation which essentially attempts to solve the inverse problem. This approach aims at enhancing the content of existing spectra databases with computed spectra of known molecular structures which have no available experimental spectra. Essentially this approach seeks to close the gap between spectral and structural databases. In silico fragmentation tools predict the fragmentation process either relying on fragmentation rules or using combinatorial/optimization-based approaches or employing machine learning methodologies^6,9,10^. Fragment prediction methods have been especially successful for predicting spectra of peptides, however, fragmentation of small molecules into ions is a more stochastic process that is especially challenging to predict^6^.

A more direct approach to the structure elucidation problem would be to reconstruct the underlying chemical structures given spectra features. Such an undertaking though is computationally challenging as it requires the generation of a molecular structure. Indeed, this approach is performed as a two-step process to circumvent the need for generating molecular structures: A machine learning model is used to map the spectrum to an intermediate vector representation such as a molecular fingerprint. Once the fingerprint is obtained then it is matched against the content of structural databases in order to identify candidate molecular structures with similar fingerprints^11,12^. This method though will also fail for molecules that are not present in the structural database and especially for novel molecules. A more direct association of spectra features with molecular structures through a rule-based approach has also been explored^13^. More specifically, this approach extracts rules, that associate spectra features with substructures, from spectra databases aiming at a partial structure identification.

An additional concept that has been introduced to facilitate the interpretation of mass spectra, and subsequently structure identification, is that of fragmentation trees^6,14^. A fragmentation tree is derived computationally from tandem mass spectra using optimization algorithms such that its nodes correspond to fragments or precursor ions and the edges correspond to fragmentation reactions. Fragmentation trees have various uses such as identifying the molecular formula and clustering molecules by aligning fragmentation trees^15^. They have also been used for the prediction of molecular fingerprints that are subsequently used to search structural databases^16,17^. The information in a mass spectrum is thought to be insufficient to explain the fragmentation process by itself while the fragmentation tree provides complementary information by elucidating the dependencies between the mass peaks^6^. However, fragmentation trees are expensive to compute and often approximations are preferred for practical applications.

A more thorough review of existing methodologies for metabolite identification, including in silico fragmentation tools, fingerprint prediction, and fragmentation trees, was recently presented by Nguyen et al. with a focus on machine learning (ML) approaches^6^. It should be noted here that early ML-based approaches were built on shallow ML models, such as Support Vector Machines (SVMs) and Random Forests (RFs), applied either on features extracted from the mass spectra or the fragmentation trees, and also kernel-based methods to determine similarity between either spectra or fragmentation trees. However, lately, there is a growing interest in exploring Deep Learning (DL) architectures for the development of computation tools to support structure elucidation. There have been efforts to learn spectra embeddings that can be subsequently used to assess spectral similarity when searching in spectral databases^12,18^. Additionally, there are DL-based methodologies for clustering spectra, either for identifying the compound class^12,19^ or for aiding medical diagnosis by differentiating between healthy and cancerous tissues^20^. Most DL-based methodologies that operate directly on spectra data are based on Convolutional Neural Networks (CNNs) representing the spectrum as a vector that indicates the intensities of each fragment mass^20–22^. The CNN attempts to automatically identify spectra features replacing the need for manual featurization. Architectures that have adopted concepts from Natural Language Processing (NLP) have also emerged representing the mass spectrum as text and the mass peaks as words^18^. Due to the limited amount of mass spectra data, different workarounds have been investigated including hybrid approaches^19^, combining statistical ML models and DL architectures, and approaches based on transfer learning^20^.

It should be noted that, at the same time, DL-based approaches are being investigated for identifying protein sequences from mass spectra in proteomics studies^21–23^. A noteworthy effort, DeepNovo, consists of an end-to-end DL architecture for de novo peptide sequencing from mass spectra^22^, which is a direct reconstruction of the peptide sequence from the mass spectra data. Structure elucidation of small molecules though is perceived as a more challenging problem due to the stochastic nature of the fragmentation process. On top of that, the structure of small molecules has a graph-like representation as opposed to the linear nature of a peptide sequence. Existing approaches essentially attempt to retrieve molecules from structure databases that have a spectrum similar to the query spectrum. This method though, cannot identify novel molecules, that is molecules whose structure currently remains unknown and therefore they do not exist in chemical databases.

In this paper, we present Spec2Mol, an end-to-end DL architecture for translating MS/MS spectra to molecular structures. Spec2Mol is intended for recommending molecular structures that can explain observed MS/MS spectra. We represent molecular structures as sequences using the SMILES notation^24^ and MS/MS spectra as vectors of fragment intensities. Spec2Mol consists of an encoder, that learns an embedding for the MS/MS spectrum, and a decoder that generates the SMILES sequences of the recommended chemical molecules. Due to the limited amount of available spectra data, our approach is based on unsupervised pre-training on a large dataset of unlabeled molecules. In particular, we pre-trained the decoder as part of an auto-encoder (AE) architecture which is trained to reconstruct a molecule through its SMILES sequence. The encoder is subsequently trained such that the spectra embeddings match the embeddings that the AE has learned. In the following sections, we discuss the data used to develop and evaluate the model, the architecture of Spec2Mol, as well as, the evaluation of the model.

The main contributions of this work are as follows:

- To our knowledge, this is the first approach for generating potential molecular structures from mass spectrometry data that is not based solely on database retrieval.
- Our method can facilitate database retrieval and additionally de novo molecular structure recommendation.
- Our approach takes advantage of large datasets of unlabeled molecules using unsupervised pre-training.
- We introduce metrics to assess the similarity of the generated molecules with the reference ones and we perform a comparative evaluation with a widely accepted method that makes use of additional information, that is fragmentation trees.

## Results and discussion

### Reconstruction accuracy of the autoencoder

As a sanity check, we evaluated the ability of the pre-trained AE to reconstruct the SMILES of the molecules in the testing set of the spectra dataset. This is performed by comparing the canonicalized input SMILES and the canonicalized output SMILES and evaluating whether there is an exact match between the two. The autoencoder is trained by minimizing the mean reconstruction error on a single-character level for each input sequence. Therefore, the reconstruction accuracy is estimated on a single-character level, by comparing the correct character in the target sequence with the most probable character in the decoder RNN’s output at each position. It should be noted, that the reconstructed SMILES, as well as neural fingerprints derived from SMILES^25–27^, has been successfully used in similarity search and have been found to be more informative, when compared to molecular fingerprints.

The AE was able to correctly reconstruct the SMILES sequence for about 93.3% of the NIST molecules. This is very close to the reconstruction rate of the AE on a held-out test set which was 94.95%. This demonstrates that the pre-trained model has been trained on a diverse set of molecules and therefore it is able to handle the large variability of the molecules in the NIST dataset.

### Spec2Mol performance evaluation

Spec2Mol generates a set of recommended molecular structures given MS/MS spectra. Our evaluation focuses on assessing the similarity between the generated structures and the reference molecular structure from the NIST dataset. We recall here that the information in an MS/MS spectrum may not be sufficient to fully reconstruct the molecular structure. It is possible that more than one molecular structures may explain a given spectrum. For that reason our analysis has been focused on assessing whether the model has learned to identify key features in the molecular structure from the mass spectra rather than identifying the exact same structure with the reference molecule from the NIST dataset.

For the evaluation of the model, we first perform a coarse-level comparison taking into account physicochemical properties and more specifically the molecular weight and the element composition of the molecule. Next, we assess molecular similarity at the substructure level. In particular, we compute the fingerprint similarity as well as the maximum common substructure between the generated structures and the reference structure. The specifications for each metric are given below. We evaluate the overall performance in the entire test set as well as the performance of the model when not all four required spectra are available as input. Additionally, we assess the contribution of each of the two strategies for generating the recommended structures.

- **Physiochemical attributes**: A property of special interest is the molecular weight since it is directly reflected in the mass spectrum. In particular, the spectra indicates the mass of the fragments and therefore the mass of the original, non-fragmented, molecule can be approximated more easily given the mass spectra as opposed to determining the composition or the structure of the molecule. We record the difference between the molecular weight of the generated structures and the reference structure and we report the relative average-minimum difference, that is, the average-minimum difference over all the predicted structures divided by the average molecular weight of the reference structures (${{{{{{{{\rm{DMW}}}}}}}}}_{\min }$). We also report the average-average difference over all the predicted structures divided by the average molecular weight of the reference structures (DMW_avg_). Additionally, we also evaluate whether the model is able to identify the element composition of the molecule. In particular, we assess whether the atom species that are present in the reference molecule have been identified in the predicted structures ignoring the numbers of atoms for each atom species. More specifically, for each atom species we report sensitivity and specificity for detecting the presence of this species. In order to account for discrepancies in the number of atoms per atom species, we also report the difference between the molecular formulas of the predicted structures and the reference structure (DMF). We define the distance between two molecular formulas as the number of atoms that differ between two molecules when accounting for the atom species and the number of atoms for each species (without including hydrogen atoms). We report the minimum distance over all predictions divided by the average number of heavy atoms (${{{{{{{{\rm{DMF}}}}}}}}}_{\min }$) as well as the average distance over all predictions divided by the average number of heavy atoms (DMF_avg_). The exact mathematical formulas for the calculation of the DMW and DMF are provided in the supplementary material (Supplementary Methods 3).
- **Fingerprint similarity:** Fingerprints are vector representations of chemical molecules, which indicate the presence of certain substructures in the molecule, and are widely used as an efficient way to judge similarity between molecules^28^. We extracted fingerprint representations based on the Morgan algorithm^29^ using the RDKit toolkit^30^ and used the cosine coefficient to assess similarity (Fngp_cosine_). The Morgan fingerprints are computed for radius 2 and 1024 bits. We report the maximum fingerprint similarity among all model predictions when compared with the reference structure as well as the average similarity of all predicted structures.
- **Maximum common substructure (MCS)**: We computed the MCS between two molecular structures using the RDKit toolkit^30^ with the following constraints: the substructure match respects the atom species, the bond orders, as well as the ring bonds, that is ring bonds are only matched to ring bonds. From the computed MCS we extracted the following three metrics: i) MCS ratio, ii) MCS Tanimoto, and iii) overlap coefficient, which are defined as follows, respectively: ${{{{{{{\rm{MC{S}}}}}}}_{ratio}=\frac{{a}_{MCS}}{{a}_{r}}}}$, ${{{{{{{\rm{MC{S}}}}}}}_{tan}=\frac{{a}_{MCS}}{{a}_{r}+{a}_{p}-{a}_{MCS}}}}$, ${{{{{{{\rm{MC{S}}}}}}}_{ovrlp}=\frac{{a}_{MCS}}{min({a}_{r},{a}_{p})}}}$, where a_MCS_ denotes the number of atoms in the MCS, a_r_ the number of atoms in the reference compound, and a_p_ the number of atoms in the predicted compound. For each metric, we report the maximum value as well as the average value over all predictions.

Table 1 summarizes the evaluation of the effect of missing data in the predictions. More specifically, we present the evaluation metrics on four different partitions of the test-set depending on the number of the available spectra. We recall that the input to the model consists of four different spectra obtained through different specifications. However, not all molecules in the dataset have all four spectra available. Our results indicate that missing only one spectrum does not severely impact performance, but performance starts to degrade when less than three spectra are available. This is expected as the number of spectral peaks that will be observed in one spectrum (or two) most likely will not be adequate to reconstruct the molecular structure. It should be noted though that other factors, such as the molecular size, are also potentially contributing to the variability observed among the different subsets of the test-set. The set of molecules with three available spectra for example, includes molecules that on average have smaller molecular weight and shorter SMILES representation. The model appears to have the highest performance on this subset of the test-set since reconstructing shorter SMILES is expected to be less of a challenge for the decoder. The evaluation of the model on the training set is presented in the supplementary material (Supplementary Note 1, Table S3).

**Table 1:** Effect of missing spectra in the model input.

metric		full dataset	4 spectra	3 spectra	2 spectra	1 spectrum
# test cases		1000	413	65	483	39
Avg. MW		275.3	287.5	242.6	267.4	300.3
Avg. SMILES length		34.5	37.0	28.5	32.5	43.6
correct molecules (*↑*)	(%)	7.0	9.2	15.2	4.1	5.1
correct formulas (*↑*)	(%)	39.3	45.1	46.9	34.8	20.5
DMW_%_ (*↓*)	Min	2.3	1.6	0.5	2.4	9.5
	Avg	6.3	5.5	3.9	6.6	14.6
DMF_%_ (*↓*)	Min	9.2	6.5	8.1	10.8	21.1
	Avg	21.7	17.8	24.5	24.0	32.9
Fngp_cosine_ (*↑*)	Max	0.53	0.56	0.57	0.50	0.45
	Avg	0.36	0.39	0.38	0.34	0.31
MCS_ratio_ (*↑*)	Max	0.68	0.70	0.72	0.66	0.57
	Avg	0.51	0.53	0.55	0.50	0.43
${{{{{{{{\rm{MCS}}}}}}}}}_{\tan }$ (*↑*)	Max	0.55	0.58	0.60	0.53	0.44
	Avg	0.38	0.39	0.41	0.36	0.30
MCS_coef_ (*↑*)	Max	0.71	0.73	0.74	0.69	0.63
	Avg	0.54	0.55	0.58	0.53	0.48

Evaluation metrics when considering the entire test set and the test-data partitions that have available all 4, only 3, only 2, and only 1 spectrum. The arrows show the desired trend for each metric.

Next, we evaluate the effect of the strategy that is used to generate the recommended molecules. The analysis is shown in Table 2. We recall that the recommended structures are obtained either directly through decoding the computed embeddings or indirectly by identifying the closest embeddings from the pre-trained dataset. In particular, we are comparing the top-20 predictions, as ranked using the molecular weight criterion, through (i) only the direct strategy, (ii) only the indirect strategy, and, (iii) the two strategies combined. According to the results, the indirect approach, which generates molecules through decoding the closest embeddings from the pre-trained dataset appears to have a larger contribution to the effectiveness of the method to generate relevant structures. However, combining the two strategies appears to slightly improve performance.

**Table 2:** Effect of the molecule generation strategy.

Metric		Direct	Indirect	Combined
Correct molecules (*↑*)	(%)	0.8	6.9	7.0
Correct formulas (*↑*)	(%)	26.1	28.0	39.3
DMW_%_ (*↓*)	min	3.1	4.4	2.3
	avg	11.6	9.3	6.3
DMF_%_ (*↓*)	min	10.4	11.9	9.2
	avg	24.2	22.4	21.7
Fngp_cosine_ (*↑*)	max	0.46	0.53	0.53
	avg	0.33	0.36	0.36
MCS_ratio_ (*↑*)	max	0.65	0.66	0.68
	avg	0.50	0.51	0.51
${{{{{{{{\rm{MCS}}}}}}}}}_{\tan }$ (*↑*)	max	0.50	0.55	0.55
	avg	0.34	0.38	0.38
MCS_coef_ (*↑*)	max	0.68	0.71	0.71
	avg	0.53	0.56	0.54

Comparative evaluation of the top-20 predictions using the direct strategy, the indirect strategy, and the two strategies combined. The arrows show the desired trend for each metric.

Overall, the results illustrate that the predicted structures have a molecular weight that is significantly close to the molecular weight of the reference compound. This is not surprising as the generated molecules are ranked based on the molecular weight. The molecular formula though seems to also be considered close to the reference one. The model was able to retrieve the exact structure for a small percentage of the test cases (7%) while it identified the exact molecular formula for a considerably larger percentage (26%). The performance of the model was significantly better when at least 3 out of the 4 input spectra were available.

Regarding the structural similarity between the predicted structures and the reference structure, the obtained values for the respective metrics demonstrate that the structures share common substructures. More specifically, the metrics that are based on the MCS between the reference and the predicted structures indicate that the common substructure is, on average, nearly 70% of the size of the reference structure for the closest structure and more than 50% for the average prediction. This result is in agreement with the high correlation between the molecular fingerprints.

Regarding the ability of the model to identify the presence of each atom species in the molecular structure, it varies significantly and it correlates with the frequency of each atom species in the training dataset, as it is shown in Table 3. More specifically, the model has very high sensitivity for nitrogen (N) and oxygen (O) which are the most common atom species in the dataset (excluding carbon which is not included in this analysis as it is present in all molecules). However, the specificity for oxygen is significantly lower than that of nitrogen which means that there is a significant number of false positives for oxygen compared to nitrogen. Regarding the rare atom species, the opposite phenomenon is observed: specificity is significantly high while sensitivity is low. This means that for the rare species there is a very small number of false positives which is expected as these atoms are under-represented in the training set. However, sensitivity is at least 0.5 for all atoms, which shows that the model is able to capture the presence of rare atoms quite well considering that some atom species are severely under-represented in the training set.

**Table 3:** Sensitivity and specificity for detecting the presence of each atom species in the entire test set, having as reference the frequency of each species in the training spectra dataset.

	O	N	S	Cl	F	Br	P	I
Sensitivity	0.94	0.86	0.50	0.68	0.48	0.79	0.53	0.51
Specificity	0.50	0.76	0.96	0.91	0.92	0.98	0.99	0.99
Frequency (%)	85.4	71.5	18.4	15.2	11.5	7.5	2.5	1.4

Finally, we investigated the effect of the molecular weight as well as the presence of heteroatoms on the ability of the model to identify the exact structure or the exact molecular formula. More specifically, we divided the test set molecules into those that have molecular weight (MW) less than 300Da and those that have molecular weight greater than or equal to 300Da (the average molecular weight in the test set is 275Da). Furthermore, we created four categories based on the presence of heteroatoms: (1) molecules that have only C and O, (2) molecules in which N is present, (3) molecules in which S is present, and, (4) molecules in which a halogen (one of Br, Cl, F, I) is present. Table 4 summarizes this analysis. The model is able to identify the atom species and atom counts for almost half of the molecules (45.4%) with MW less than 300Da and for more than 60% of the molecules that contain only C and O (63.6%). The higher molecular weight as well as the presence of atoms that are under-represented in the training set (S and halogens) degrades the ability of the model to identify the molecular structure or formula.

**Table 4:** Effect of molecular weight and presence of heteroatoms.

	MW < 300	MW ≥ 300	Only C and O	N present	S present	Halogen present
Number of cases	668	332	184	769	199	318
Exact structure (%)	8.5	3.9	9.8	6.1	5.5	5.7
Exact formula (%)	45.4	27.1	63.6	34.1	23.6	25.8

**Fig. 2:**
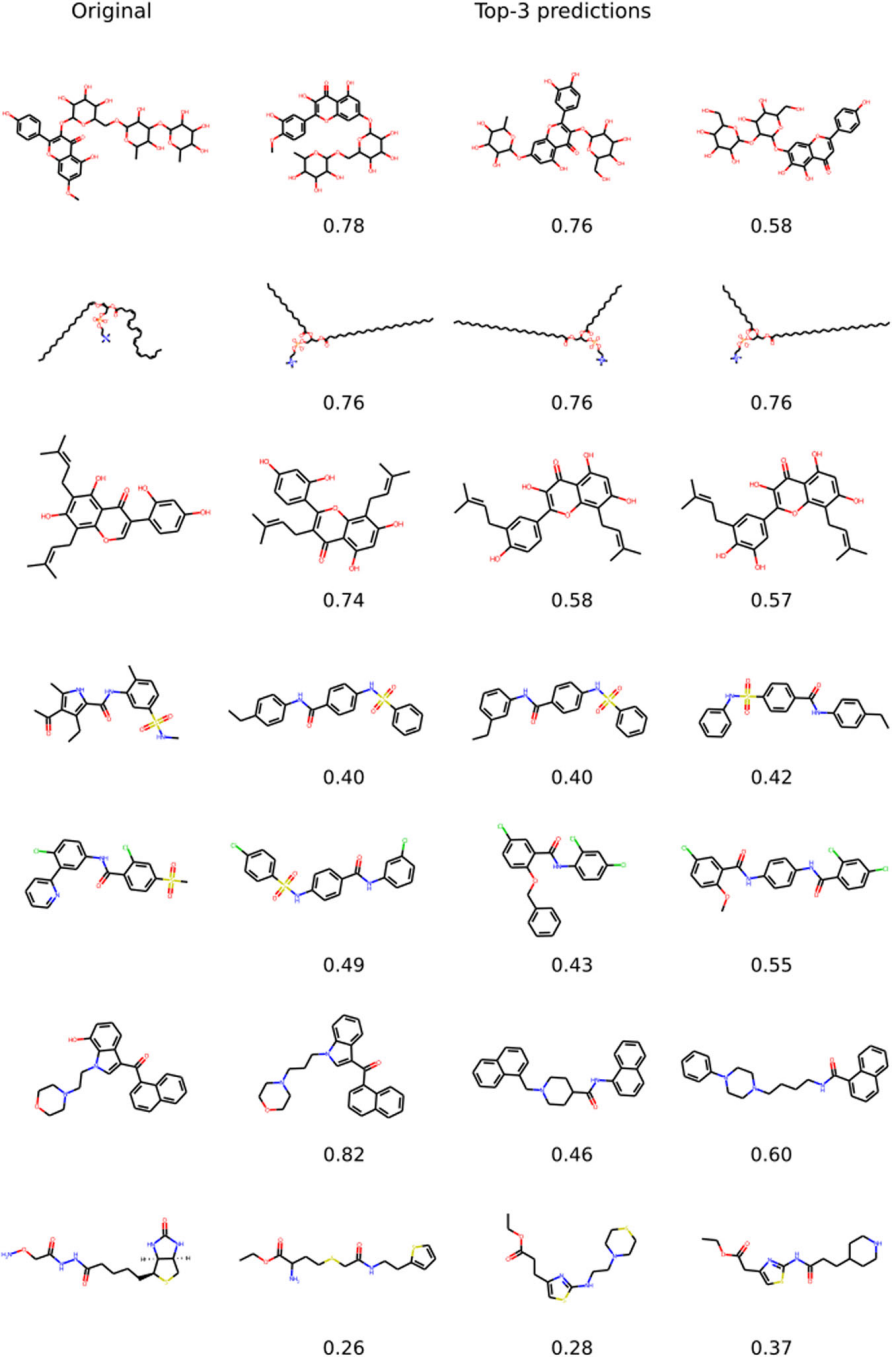
Examples of cases where Spec2Mol successfully identified key substructures. Examples of the most likely predicted structures from Spec2Mol along with the cosine similarity values with respect to the original reference structures.

Figure 2 shows a few examples of successful cases with the model correctly identifying key substructures such as rings and long chains and the presence of rare atoms and functional groups. Given the vast space of possible molecular structures, these cases demonstrate that the model has indeed learned to associate spectra features with molecular structures.

**Fig. 3:**
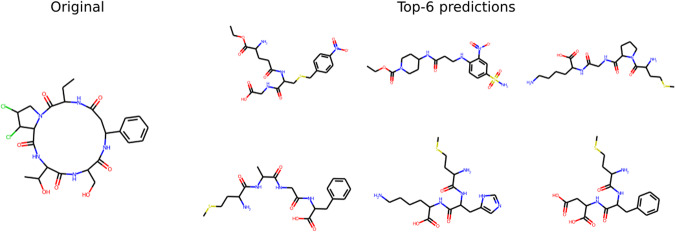
A case where Spec2Mol did not identify relevant structures. An example where Spec2Mol failed to identify a similar structure for a reference compound containing a large ring.

We also identify two general scenarios where the model has difficulty in predicting relevant structures: (1) Molecules with large rings and (2) Molecules that have poor-quality spectra. An example of the first case is illustrated in Fig. 3. We believe this is because molecules with large rings are significantly under-represented in the dataset that was used to pre-train the decoder. Also, it is hard to generate a valid SMILES sequence for molecules with very large rings. Regarding the second case of poor quality input spectra, it includes cases where there is a very small number of peaks in the spectra and therefore not adequate information to reconstruct the SMILES sequence.

### Comparative evaluation

In order to perform a comparative evaluation, we have used SIRIUS 4^31^, which offers multiple functions including chemical formula, as well as molecular structure, identification from mass spectra. SIRIUS’ structure elucidation method, called CSI:FingerID, is a database retrieval method^16^. It relies on Support Vector Machines (SVMs) for predicting a molecular fingerprint and subsequently compares the predicted fingerprint against those of a reference database in order to identify candidate structures. The input to the SVM is the MS/MS spectrum along with the corresponding computed fragmentation tree. CSI:FingerID has shown superior performance when compared to other existing tools for the automatic identification of molecular structures from spectra data. In particular, it was the best-performing method in the Critical Assessment of Small Molecule Identification (CASMI) contest for 2016 and 2017^31^. However, the performance of this method degrades significantly for cases that are not covered in the training set^31^. Additionally, the dependence of CSI:FingerID on fragmentation tree data adds significantly to the running time of this method.

We run SIRIUS on the same test set we developed for evaluating Spec2Mol. As input, we provided SIRIUS with the positive mode spectra (that is [M+H]+ at low and high energy) as they were selected for Spec2Mol. The spectra from negative ions were not used since a single run for SIRIUS accepts spectra from a single precursor which may be obtained through different energies. As 53 test cases out of the 1000 cases of the test set did not have any positive mode spectra and therefore the test set used for the comparison consists of 947 cases. As a side note, SIRIUS performs structure elucidation after identifying the molecular formula. The number of molecular formulas to be explored is one of the parameters of the tool which we set to 10. An additional parameter is the reference database which we set to PubChem, which is the largest available source offered by SIRIUS. Finally, SIRIUS allows the user to define the set of chemical elements to be considered when performing the search which we set to C, H, O, N, S, Cl, F, Br, P, and I. It should be noted that expanding the pre-defined set of atoms (C, H, N, O, P, S) to account for more rare atoms, which were present in the NIST dataset, significantly increased the running time.

On the test set of 947 cases, SIRIUS found the correct formula for about 98% of the test cases while it found the correct structure for about 67%. For 6 cases out of 947, SIRIUS did not return any structures. It should be highlighted that the CSI:FingerID method from SIRIUS has been trained on the NIST dataset (NIST v17). As it is discussed in the original study on the SIRIUS tool, the presence of spectra for a given test structure in the training set can significantly boost performance even if the spectra that are used when testing is not the exact same spectra as the ones used in training^31^.

The comparative evaluation between SIRIUS and Spec2Mol was performed on the cases where SIRIUS failed to find the exact molecular structure. Since Spec2Mol is intended for recommending potential molecular structures given mass spectra, our intention here is to evaluate how relevant the recommendations are, when compared to a widely accepted and state-of-the-art method like SIRIUS. By focusing our comparison on the cases where SIRIUS did not find an exact match, we are essentially evaluating the relevance of the recommended structures when an exact match is not found, which points to the case of novel molecules. In particular, we compared SIRIUS and Spec2Mol on the 307 cases, for which SIRIUS failed to find an exact match, using the metrics based on fingerprint similarity and MCS. It should be noted here that failure to identify the exact structure includes cases where SIRIUS either did not return any structure as well as cases where the reference structure was not among the predicted structures. The results are summarized in Table 5. The comparison of the full test set (including cases where SIRIUS found the exact structure) is provided in the supplementary material (Supplementary Note 2, Table S4). According to our analysis, the structures recommended by Spec2Mol are at least as relevant as the ones recommended by SIRIUS. More specifically, Spec2Mol achieved slightly better cosine similarity for the closest structure, while almost all metrics based on the MCS are improved in the case of Spec2Mol. This outcome is especially interesting and encouraging, given that Spec2Mol is an end-to-end approach that does not take into account any prior knowledge. Spec2Mol generates potential molecular structures by solely looking at raw MS/MS spectra. On the other hand, the combination of CSI:FingerID and SIRIUS attempts to retrieve the exact molecular structure from a reference database taking as input the computed fragmentation tree on top of the raw mass spectra. It should be stressed that a direct comparison of the two methods is not possible since they differ significantly: CSI:FingerID uses predicted fingerprints from the MS/MS spectrum of an unknown compound to find the best match against a chemical structure database, while Spec2Mol aims for de-novo generation of potential molecular structures rather than attempting the best match retrieval from a database. Therefore, Spec2Mol is useful in situations where a reference database is not available or CSI-FingerID cannot find an exact match. For that reason, the comparison is performed on the cases where CSI-FingerID failed to identify the exact structure, and the metrics used aim at evaluating molecular similarity rather than exact matches.

**Table 5:** Comparative evaluation between SIRIUS and Spec2Mol, based on structural similarity between the recommended structures and the reference structure, on the subset of the test set where SIRIUS failed to identify an exact match.

Method		Fngp_cosine_	MCS_ratio_	${{{{{{{{\rm{MCS}}}}}}}}}_{\tan }$	MCS_coef_
SIRIUS	Max	0.49	0.65	0.54	0.66
	Avg	0.33	0.49	0.35	0.49
Spec2Mol	Max	0.49	0.66	0.53	0.69
	Avg	0.34	0.50	0.36	0.53

Still, the outcome of our comparative evaluation demonstrates that the molecular structures generated by Spec2Mol are at least as successful as the ones obtained by state-of-the-art tools when considering novel molecules despite the fact that Spec2Mol relies solely on raw MS/MS spectra.

## Conclusions

Elucidating the structure of chemical compounds is a fundamental, but cumbersome, task in metabolomics studies, as well as in chemical analysis in various domains including drug development and forensics analysis. The available computational tools for aiding structure elucidation are based on fragment annotation and database retrieval methods. This approach fails to identify molecules that are not present in the reference database which, in practice, may correspond to a considerably large percentage of the query spectra. We have developed Spec2Mol, an end-to-end deep learning architecture for directly generating molecular structures (as SMILES sequences) from the input MS/MS spectra. Spec2Mol is based on an encoder-decoder architecture that generates molecular SMILES sequences, given mass spectra. While the proposed architecture supports the retrieval of molecules from a database that best matches the input spectra, it can also generate new molecules that have not been seen before in any dataset. Our analysis demonstrates that the recommended molecules are structurally, and physiochemically, similar to the reference compounds, suggesting that the latent space has indeed learned informative associations between the spectra and the structural features. When compared to an existing method that depends on the fragmentation tree annotation, on top of the raw spectra for molecule identification, Spec2Mol performed on par for the task of recommending potential molecular structures. Our results indicate that the proposed approach of recommending de-novo molecules directly from input MS spectra provides critical insights into the characteristics of the underlying molecular structure, and, can complement existing tools especially when the current tools fail to identify the right molecule from existing databases. We speculate that incorporating prior knowledge in the model, for example in the form of fragmentation trees, can further boost the performance of the proposed method. Further, even though the main focus of our work is on de-novo generation of molecules given an input spectrum, the indirect method proposed by our paper can be extended to identify the correct molecule from a library of a plausible set of molecules, similar to the work proposed by Lim et al.^32^. A substructure-constrained similarity search or the nearest neighbor search on the embeddings of the molecule library with the spectra embedding as a query can be used to identify the best candidates from a relevant library.

## Methodology

Spec2Mol consists of an encoder that learns spectra embeddings and a pre-trained decoder, which has been trained as part of an autoencoder architecture. The autoencoder has been trained on a large set of molecules (molecule dataset discussed in section Molecule dataset), while the encoder has been trained on a set of molecules for which MS/MS data are available (spectral dataset discussed in section Spectral dataset).

### Datasets

#### Molecule dataset

The autoencoder, from which the Spec2Mol decoder has been derived, was pre-trained on about 135 million molecules which were sourced from the PubChem^33^ and ZINC-12^34^ datasets. The structures of these molecules are represented using the SMILES notation^24^. Stereochemistry information was not indicated in the SMILES representation. The reason for not accounting for stereochemistry is that, in the subsequent task of spectra translation, recovering stereochemistry information from the mass spectra is especially challenging or possibly even impossible and therefore it is out of the scope of this work.

#### Spectral dataset

The mass spectra data for training the encoder has been derived from the NIST Tandem Mass Spectral Library 2020 which is a commercial dataset of more than 1M spectra obtained from more than 30K compounds^35,36^. The largest percentage of the NIST dataset (60%) corresponds to metabolites (6K human metabolites and 8K plant metabolites) while a significant amount of the data is drugs (20%). The rest corresponds to peptides, lipids, forensics, surfactants/contaminants and sugars/glycans. The dataset contains low and high resolution MS/MS spectra, obtained through different fragmentation techniques. Each molecule in the dataset may be associated with more than one spectra which may be obtained through different experimental conditions, that is, different fragmentation instrument, precursor ion, ionization mode, collision energy or fragmentation level (MS2, MS3 or MS4). Statistics of the dataset regarding common molecular properties (e.g. molecular weight, number of atoms and number of rings), as well as the atom species coverage, are presented in the supplementary material (Supplementary Methods 1, S1.2, Tables S1-S2).

### Data processing and representation

In order to minimize variations in the spectra data, due to differences in the experimental conditions, we chose to keep certain variables in the dataset fixed. Details on the filtering process that we followed for constructing the spectral dataset are provided in the supplementary material (Supplementary Methods 1, S1.1). More importantly, we used only the spectra that are obtained through the most common precursor ions, that is [M+H]+ and [M-H]-. For each precursor ion, we used two spectra, one obtained using low collision energy (35% NCE) and one with high collision energy (130% NCE). Therefore, each instance in the dataset we constructed is characterized by four MS/MS spectra derived from two different precursor ions and two energy levels. The four spectra constitute the input to the spectra encoder as described in paragraph 3.2. It should be highlighted though, that not all molecules in the NIST dataset have experimental data for the specific precursors and energy levels. However, we have allowed cases with missing data in the dataset and the missing spectra are represented as empty spectra, that is spectra with no peaks, in an attempt to develop a model that is robust to missing data. Therefore, the model is being trained and evaluated on cases that may not have available all four spectra.

#### Data representation

We represent each MS/MS spectrum as a vector in which each bit corresponds to a specific mass-over-charge (*m*/*z*) value, representing the *m*/*z* value of the recorded fragments, while the value of each bit corresponds to the intensity, or otherwise frequency, of the fragments that have been recorded with that specific mass-over-charge value. We have normalized the intensity values by dividing with the maximum intensity over all the vector bits of a given spectrum. More details on the representation of the MS/MS spectra are provided in the supplementary material (Supplementary Methods 1, S1.3). Regarding the molecular structures, we represent them using canonical SMILES without indicating stereochemistry information.

#### Data augmentation

The variability in the spectra for a given molecule opens up the possibility for data augmentation. In particular, although some spectra from the same molecule may differ significantly, as shown in Fig. 1, in many cases the obtained spectra are closely related. One such case is when the collision energies that are being used are relatively close.

In order to augment the dataset, for each instance in the training set we are creating an additional training instance by slightly perturbing the collision energy in all four spectra. In particular, each spectrum, out of the four spectra that are used to represent an instance in the dataset, is replaced with a spectrum that has the closest collision energy in the dataset while all other parameters (precursor ion, instrument) are shared. More information is provided in the supplementary material (Supplementary Methods 1, S1.4).

#### Data partition

After the data filtering process, the acquired dataset consists of 23K molecules, each one of them is associated with four MS/MS spectra, or more precisely, up to four MS/MS spectra given that there are cases with missing spectra. This dataset was partitioned into a training, a validation and a test set with the validation and test set having about 1K molecules each. For the test set specifically, we used fingerprint similarity, based on the Tanimoto coefficient^28^, in order to ensure that no test molecule is either in the train or in the validation set. The validation set was used to select the model hyper-parameters and the test set was used to evaluate the performance of the model.

### Spec2Mol architecture

**Fig. 4:**
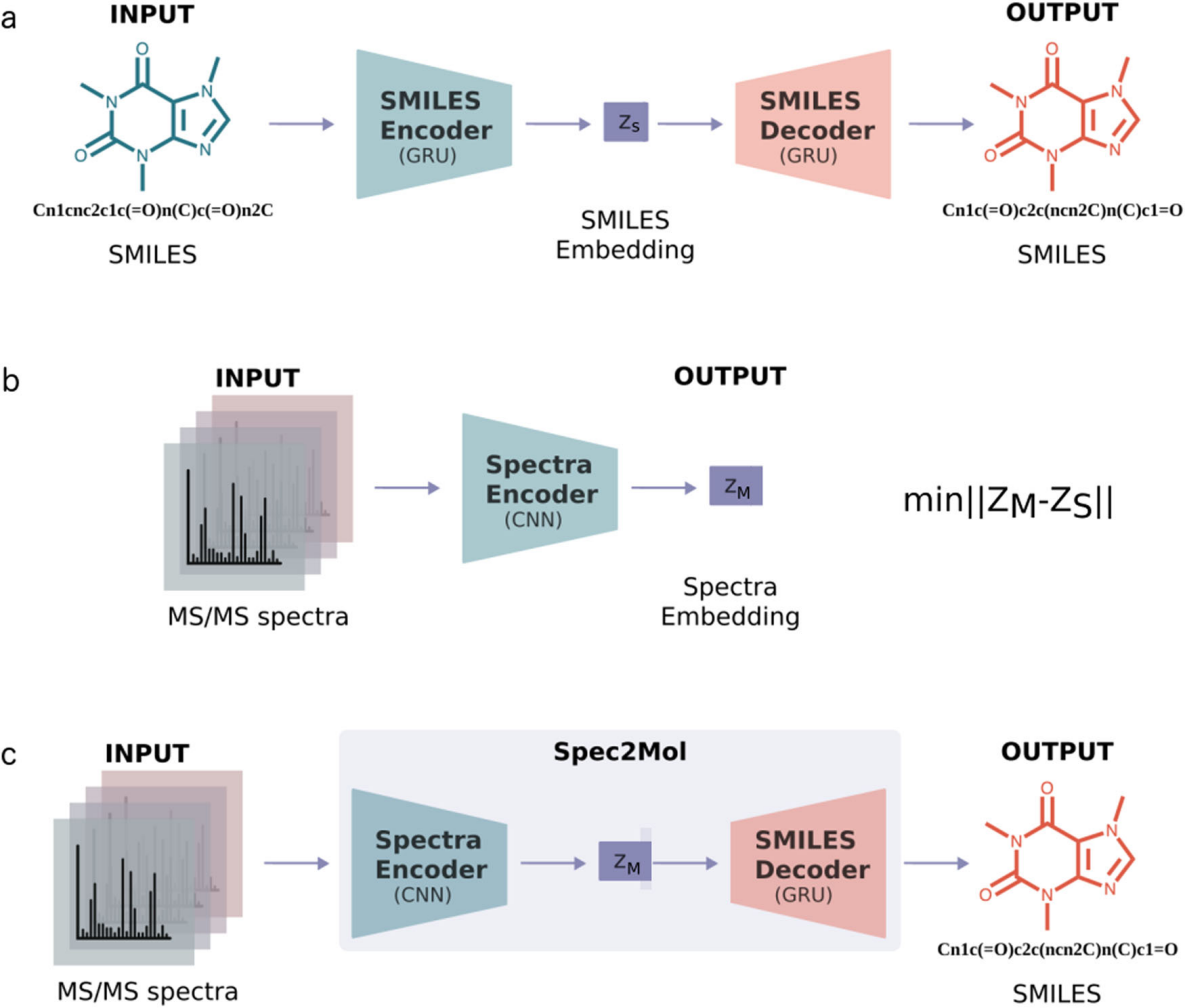
Spec2Mol architecture. The Spec2Mol model consists of a spectra encoder and a SMILES decoder which have been trained separately but share the same embedding space. **a** The AE is pre-trained to translate from a random SMILES to the canonical SMILES string. **b** The spectra encoder is trained to learn the same embedding as the SMILES encoder. **c** During inference, the spectra encoder and the SMILES decoder of the pre-trained model are used to translate spectra into molecular structures.

Spec2Mol uses an encoder-decoder architecture for recommending molecular structures from MS/MS spectra. The Spec2Mol encoder generates spectra embeddings while the decoder reconstructs the SMILES sequence from a spectra embedding. The encoder and the decoder have been trained separately as it is shown in Fig. 4. First, the decoder is trained as part of an autoencoder architecture for reconstructing the SMILES sequence from a SMILES embedding. Next, the spectra encoder is trained such that the learnt spectra embeddings match the corresponding SMILES embeddings. Finally, for making inference on unseen cases, Spec2Mol uses the spectra encoder to obtain the spectra embedding which is subsequently used in order to decode potentially novel molecules and also to retrieve molecules from the pre-training dataset.

The specifications for training each model are given in the following paragraphs while more details on the architectures of the models, hyperparameters, and training parameters are provided in the supplementary material (Supplementary Methods 2).

### Pre-training the AE on chemical structures

The autoencoder is trained on a translation task where a randomized input SMILES is translated into its corresponding canonical SMILES, similar to the work of Winter et al^25^. The encoder and the decoder of the AE are both based on gated recurrent units (GRU) which is a variation of the standard long short term memory (LSTM) models, that are commonly used for learning sequence representations, with fewer parameters. The details regarding the autoencoder architecture are in the supplementary material (Supplementary Methods 2, S2.1).

### Training the spectra encoder

The spectra encoder is trained in a supervised manner such that the learned spectra embeddings are the same as the SMILES embeddings that the AE has learned. More specifically, the input of the spectra encoder consists of the four spectra that have been pre-selected to represent each molecule. The spectra encoder is based on 1-D CNNs and in particular consists of two 1-D CNN layers and two fully connected layers. The four spectra are represented as 4 discrete vectors which are fed into the 1-D CNN as data from four different channels. Each channel corresponds to a specific precursor ([M+H]+ or [M-H]-) and energy level (low or high). If any of the required four spectra are not available, then the input to the respective channel is an all-zeros vector. The output of the spectra encoder is a 1-D vector which is the latent representation of the spectra in the embedding space. The model is trained such that the distance (root mean square error) between the latent representation that is learned by the spectra encoder and the latent representation that is obtained from the pre-trained SMILES encoder is minimized. Details regarding the architecture and training of the spectra encoder are provided in the supplementary material (Supplementary Methods 2, S2.2).

### Recommending molecular structures for unseen spectra

Spec2Mol provides as output molecular structures that can potentially explain the observed spectra peaks. The recommended molecules for unseen spectra are obtained using two strategies: a direct and an indirect molecule generation strategy. The direct molecule generation strategy generates molecular structures using the SMILES decoder from the computed MS/MS embedding. Multiple SMILES are generated for each MS/MS embedding using a pure sampling strategy^37^, and subsequently filtered in order to retain only the valid ones, i.e., the sequences that are in accordance with the SMILES syntax. The indirect strategy retrieves molecular structures from the dataset that was used for pre-training the AE based on the distance in the embedding space. More specifically, for each MS/MS embedding we find the closest embeddings from the pool of molecules used to pre-train the AE and decode those embeddings into SMILES sequences.

The predicted molecules obtained through these two strategies are combined and ranked based on their discrepancy from the expected molecular weight. The molecular weight of the underlying chemical structure is easily inferred from the mass spectrum and therefore in this work we consider it as known. The molecular structures that have molecular weight closer to the reference weight are highly ranked. The top-20 ranked predictions are returned to the user.

## Supplementary information

Supplementary material

## Data Availability

The spectra dataset used for training and evaluating the model cannot be made publicly available as it is a commercial dataset.

## Code availability

The code is available at https://github.com/KavrakiLab/Spec2Mol.
