# Supplementary material for: An end-to-end deep learning framework for translating mass spectra to de-novo molecules
Source: Commun Chem. 2023 Jun 23;6:132. doi: 10.1038/s42004-023-00932-3 (PMC10290119; doi:10.1038/s42004-023-00932-3)
Supplement: Supplementary file 1 — Supplementary material [file 42004_2023_932_MOESM1_ESM.pdf]

An end-to-end deep learning framework for translating mass spectra to de-novo molecules

Eleni E. Litsa[a], **Vijil Chenthamarakshan**[b], **Payel Das**[*b], **and Lydia E. Kavraki**[*a]

[a]Department of Computer Science, Rice University, Houston, TX
[b]IBM Research AI, IBM Thomas J. Watson Research Center, Yorktown Heights, NY 10598
[*]daspa@us.ibm.com, kavraki@rice.edu

SUPPLEMENTARY INFORMATION

Supplementary Methods 1: Spectra dataset information

S1.1 Data filtering

The spectra dataset that was used for the development of the spectra encoder was derived from the NIST dataset[1,2] as follows:

- We used only the high resolution MS/MS spectra and more specifically we used the spectra that are obtained through higher-energy collision dissociation (HCD) which was the most common fragmentation method in the NIST dataset and additionally it is known to have high sensitivity and produce more fragments.
- We did not use MS3 and MS4 spectra as these were provided only for a small percentage of the data.
- Regarding the precursor ions, we retained only the most common ones, that is [M+H]+ and [M-H]-.
- For each precursor ion, we used two spectra, one obtained using low collision energy and one with high collision energy. The level for characterizing low collision energy was set to 35% NCE (Normalized Collision Energy) and for high energy it was set to 130% NCE. These values were selected because they were the most common energy levels in the NIST dataset for characterizing low and high energy, respectively. In the cases where a spectrum with energy 35% or 130% NCE was not available, we selected the spectrum that was obtained using collision energy that was closest to that level.
- We removed molecules with rare atom species, that is species that appeared in less than 30 molecules. Specifically, we excluded molecules with the following atoms: Co, Fe, Se, As, Si, B, Sn, Au, Cu.
- We did not make use of the data corresponding to peptides since the goal of this work is to identify structures of small molecules.
- We filtered out all molecules for which the retained spectra, for the selected precursor ions and energies, did not have peaks with $m/z > 500$.

Note that the AE was pre-trained on a large set of molecular structures derived from the PubChem database, while the molecules in the spectra dataset were obtained from the NIST database. Though, the molecules from the NIST dataset were not explicitly added to the training set while pre-training the AE, we found that about 91% of the NIST molecules were found in the PubChem database.

S1.2 Data statistics

The dataset that was acquired after filtering the NIST dataset (as described in S1.1), consists of 22931 molecules in total from which 961 molecules were used in the validation set for determining the hyper-parameters of the spectra encoder and 1000 molecules were set aside as a test set.

The training and validation sets combined include 21930 molecules. 62.5% of the molecules in the training and validation sets have two available spectra, 27.5% have four, 5% have one spectrum and 5% have three available spectra. The total number of spectra used for training and validation purposes is 55,935. The big majority of these spectra correspond to the [M+H]+ precursor ion. In particular, 35% of the spectra used for training and validation correspond to [M+H]+ and low energy, and 34% correspond to [M+H]+ and high energy. The spectra obtained from the precursor ion [M-H]- and low energy correspond to 16% of the total spectra in train and validation datasets and the rest 15% comes from [M-H]- and high energy.

Table S1. Statistics from molecular properties (molecular weight (MW), number of atoms, number of rings, and size of largest ring) describing the molecules from NIST that used to train the spectra encoder.

dataset		MW	atoms	rings	largest ring
	min	82.1	4	0	0
train	max	1449.6	101	12	24
	avg	274.5	18.8	2.1	5.5
	min	100.2	6	0	0
valid	max	850.0	61	8	24
	avg	275.7	18.8	2.1	5.6
	min	101.1	6	0	0
test	max	818.2	57	8	16
	avg	275.3	18.7	2.1	5.5

Table S2. Percentage of molecules in which each atom species appears.

	C	O	N	S	Cl	F	Br	P	I	B	Si
train	100	85.4	71.5	18.4	15.2	11.5	7.5	2.5	1.4	0.1	0.1
valid	100	85.3	74.5	19.8	17.1	13.0	8.6	2.0	2.0	0	0.2
test	100	89.5	76.9	19.9	13.9	12.8	8.4	1.4	2.6	0	0

S1.3 Data representation

We represent each MS/MS spectrum as a vector in which each bit corresponds to a specific mass-over-charge (m/z) value, representing the m/z value of the recorded fragments, while the value of each bit corresponds to the intensity, or otherwise frequency, of the fragments that have been recorded with that specific mass-over-charge value. For that representation, we need to specify the resolution of the mass as well as the minimum and maximum allowed mass values. More specifically, the minimum mass is set to 50 Da while the maximum mass is set to 500 Da. The resolution for the mass values is 0.01 Da. Given that our dataset is of higher resolution, that is more than 4 decimal points are available, the intensity of each bit corresponds to an aggregation of all fragments that have been recorded and have the same mass when considering two decimal points. This representation results in a vector of length 50,000. Finally, the intensity values are normalized by dividing with the maximum intensity over all the vector bits of a given spectrum. The minimum and maximum allowed mass values were selected based on the statistics of the dataset. More specifically, the minimum allowed mass corresponds to the minimum fragment mass that has been recorded over all data. Regarding the maximum allowed mass, although there are molecules in the dataset with larger recorded fragments, the percentage of molecules with fragments larger than 500 Da is very small. In general, a smaller maximum allowed mass, as well as a lower resolution, will result in a more compact and less sparse vector representation which is essential for preventing over-fitting when training the spectra encoder.

We represent the molecular structures using canonical SMILES without indicating stereochemistry information.

S1.4 Data augmentation

In order to augment the dataset, for each instance in the training set we are creating an additional training instance by slightly perturbing the collision energy in all four spectra. In particular, each spectrum, out of the four spectra that are used to represent an instance in the dataset, is replaced with a spectrum that has the closest collision energy in the dataset with the spectrum to be replaced, while all other parameters (precursor ion, instrument) are shared.

In order to avoid large deviations from the preset energy levels (35% for low energy and 130% for high energy) we perturbed only the spectra that had exactly the pre-set energy levels (we recall here that in cases where a spectrum of 35% or 130% NCE was not available, it was already replaced with the closest available in the original dataset).

Without performing data augmentation, the dataset consists of 20,970 training instances. After performing augmentation it consists of 38,504 training instances.

Supplementary Methods 2: Models' architectures & Hyperparameters

S2.1 Autoencoder

Architecture:

- Bidirectional GRU with an encoder-decoder architecture and
  - number of encoder and decoder layers: 3
  - hidden dimension for encoder and decoder: 512
  - Dimensionality of the embedding space: 512
  - nonlinearity: tanh

Training Hyperparameters:

- Batch size: 256
- Learning rate: 0.0001

Training: The autoencoder is trained as a translation model by translating from a randomized SMILES version of a molecule to its canonical version. The model is trained on 135M molecules from Pubchem and ZINC12 datasets.

The architecture of the translation model and latent dimension of 512 is similar to the one used in Winter et al.[3]

In order to make the learnt representations more meaningful, we also jointly trained a regression model to predict some molecular properties that can be calculated using the molecular structure. The regression model uses two fully connected layers with dimensions 512 and 128 and ReLU non-linearity. The properties that are predicted are: logP, molar refractivity, number of valence electrons, number of hydrogen bond donors and acceptors, Balaban's J value, topological polar surface area, drug likeliness (QED) and synthetic accessibility (SA).

S2.2 Spectra encoder

Architecture:

- Two 1-Dimensional Convolution layers, which operate on the raw spectra, with:
  - kernel dimension: 200 (both layers)
  - Padding: 100 (both layers)
  - Stride: 1 (both layers)
  - Number of channels:
    - Conv 1: Input channels 4 and output channels 4
    - Conv 2: Input channels 4 and output channels 8
- 1 dimensional batch normalization between layers
- Max pooling with kernel dimension 50 for both convolutional layers
- Two layers of fully connected neural networks which operate on the output of the convolutional layers (after applying pooling)
- Dimensionality of the embedding space: 512

Training hyper-parameters:

- Learning rate with exponential decay with initial value 0.004 and exponential decay parameter $gamma = 0.98$
- Batch size: 512
- Weight decay: 0.0001
- Number of epochs: 200

The limited number of layers in the spectra encoder is due to the limited available MS/MS spectra for training the encoder. Architectures with higher complexity were prone to overfitting.

S2.3 Implementation

The models were developed using the PyTorch[4] library.

Supplementary Methods 3: Evaluation metrics DMW and DMF

To evaluate the discrepancies between the molecular weight (MW) of the predicted molecules and the molecular weight of the reference molecule, we established the following two metrics:

- Relative average minimum MW difference: $\mathrm{DMW}_{\mathrm{min}} = \dfrac{\frac{1}{n}\sum_{i=1}^{n}\left(\min_{j=1\dots m}\{|\widehat{\mathrm{MW}}_i^{(j)} - \mathrm{MW}_i|\}\right)}{\frac{1}{n}\sum_{i=1}^{n}\mathrm{MW}_i}$
- Relative average-average MW difference: $\mathrm{DMW}_{\mathrm{avg}} = \dfrac{\frac{1}{n}\sum_{i=1}^{n}\left(\frac{1}{m}\sum_{j=1}^{m}\{|\widehat{\mathrm{MW}}_i^{(j)} - \mathrm{MW}_i|\}\right)}{\frac{1}{n}\sum_{i=1}^{n}\mathrm{MW}_i}$

where $i = 1...n$ is the index of the different molecules, while j is the index of the various predictions for a molecule. $\mathrm{MW_i}$ is the molecular weight of the reference molecule with index i, while $\widehat{\mathrm{MW}}_\mathrm{i}^{(j)}$ is the molecular weight of the molecule that corresponds to the predicted SMILES.

Similarly, for the molecular formulas (MF), we defined the following two metrics in order to evaluate discrepancies in the atom species and counts:

- Relative average-minimum MF distance: $\mathrm{DMW_{min}} = \dfrac{\frac{1}{n}\sum_{i=1}^{n}\left(\min_{j=1...m}\{\mathrm{DMF}^{(j)}\}\right)}{\frac{1}{n}\sum_{i=1}^{n}\mathrm{HA_i}}$
- Relative average-average MF distance: $\mathrm{DMF_{avg}} = \dfrac{\frac{1}{n}\sum_{i=1}^{n}\left(\frac{1}{m}\sum_{j=1}^{m}\{\mathrm{DMF}^{(j)}\}\right)}{\frac{1}{n}\sum_{i=1}^{n}\mathrm{HA_i}}$

where $\mathrm{HA_i}$ is the number of heavy atoms in the reference molecule with index i, and $\mathrm{DMF^j}$ is the number of atoms that are different between the molecular formula of the reference molecule and the prediction j, when accounting for the atom species and the number of atoms for each species (without including hydrogen atoms). For example, the distance between two molecules with molecular formulas $C_2H_4O_2$ and C_3H_6O is 2 (1 carbon atom and one oxygen atom).

Supplementary Note 1: Evaluation on train set

Table S3 shows the evaluation metrics on the training set (20,970 molecules) and training set (1,000 molecules). As shown in Table S1, the training set is a more challenging dataset, with molecules of higher molecular weight on average. This observation can explain the fact that the model has comparable performance on the two datasets although we would expect to see improved performance on the training set.

Table S3. Model evaluation on the training and testing set

metric		train	test
correct molecules (↑)	(%)	6.9	7.0
correct formulas (↑)	(%)	25.1	26.0
$\mathrm{DMW_{\%}}$ (↓)	min	1.7	1.5
	avg	6.1	5.4
$\mathrm{DMF_{\%}}$ (↓)	min	9.6	9.2
	avg	21.6	21.7
$\mathrm{MCS_{ratio}}$ (↑)	max	0.69	0.68
	avg	0.53	0.51
$\mathrm{MCS_{tan}}$ (↑)	max	0.57	0.55
	avg	0.39	0.38
$\mathrm{MCS_{coef}}$ (↑)	max	0.73	0.71
	avg	0.57	0.54

Supplementary Note 2: Comparative evaluation between Spec2Mol and SIRIUS

A comparative evaluation between Spec2Mol and CSI:FingerID from SIRIUSis presented in Table S4. The evaluation is being performed on:

- The full test set of 1000 set-aside* molecules.
- The hard test set of 307 molecules on which SIRIUS did not find an exact match.

* set aside test set for Spec2Mol (this test set is not a set-aside test set for SIRIUS).

Table S4. Comparative evaluation of structural similarity between the recommended structures and the reference structure between SIRIUS and Spec2Mol.

Test set	Method		$Fngp_{cosine}$	MCS_{ratio}	MCS_{tan}	MCS_{coef}
full test set	SIRIUS	max	0.83	0.89	0.85	0.89
		avg	0.46	0.61	0.48	0.61
	Spec2Mol	max	0.53	0.68	0.55	0.71
		avg	0.36	0.51	0.37	0.54
hard test set	SIRIUS	max	0.49	0.65	0.54	0.66
		avg	0.33	0.49	0.35	0.49
	Spec2Mol	max	0.49	0.66	0.53	0.69
		avg	0.34	0.50	0.36	0.53

Supplementary References

1. X. Yang, P. Neta, and S. Stein, "Extending a tandem mass spectral library to include ms2 spectra of fragment ions produced in-source and msn spectra," *Journal of the American Society for Mass Spectrometry*, vol. 28, p. 2280–2287, 2017.

2. "NIST 20 dataset." https://chemdata.nist.gov/dokuwiki/lib/exe/fetch.php?media=chemdata:asms2020:xiaoyu_yang_asms2020_presentation.pdf. Accessed: 2021-04-04.

3. R. Winter, F. Montanari, F. Noé, and D.-A. Clevert, "Learning continuous and data-driven molecular descriptors by translating equivalent chemical representations," *Chemical science*, vol. 10, no. 6, pp. 1692–1701, 2019.

4. A. Paszke, S. Gross, F. Massa, A. Lerer, J. Bradbury, G. Chanan, T. Killeen, Z. Lin, N. Gimelshein, L. Antiga, A. Desmaison, A. Kopf, E. Yang, Z. DeVito, M. Raison, A. Tejani, S. Chilamkurthy, B. Steiner, L. Fang, J. Bai, and S. Chintala, "Pytorch: An imperative style, high-performance deep learning library," in *Advances in Neural Information Processing Systems 32* (H. Wallach, H. Larochelle, A. Beygelzimer, F. d'Alché-Buc, E. Fox, and R. Garnett, eds.), pp. 8024–8035, Curran Associates, Inc., 2019.
